# Elevated Circulating Osteoprotegerin and Renal Dysfunction Predict 15-Year Cardiovascular and All-Cause Mortality: A Prospective Study of Elderly Women

**DOI:** 10.1371/journal.pone.0134266

**Published:** 2015-07-29

**Authors:** Joshua R. Lewis, Wai H. Lim, Thor Ueland, Germaine Wong, Kun Zhu, Ee M. Lim, Jens Bollerslev, Richard L. Prince

**Affiliations:** 1 University of Western Australia School of Medicine and Pharmacology, Sir Charles Gairdner Hospital Unit, Perth, Australia; 2 Department of Endocrinology and Diabetes, Sir Charles Gairdner Hospital, Perth, Australia; 3 Department of Renal Medicine, Sir Charles Gairdner Hospital, Perth, Australia; 4 Research Institute of Internal Medicine, Oslo University Hospital Rikshospitalet, Oslo, Norway; 5 Centre for Kidney Research, Children's Hospital at Westmead School of Public Health, Sydney Medical School, The University of Sydney, Sydney, Australia; 6 PathWest, Sir Charles Gairdner Hospital, Perth, Australia; 7 Section of Specialised Endocrinology, Department of Endocrinology, Oslo University Hospital, Rikshospitalet, and University of Oslo, Oslo, Norway; German Diabetes Center, Leibniz Center for Diabetes Research at Heinrich Heine University Duesseldorf, GERMANY

## Abstract

**Background:**

Data on the predictive role of estimated glomerular filtration rate (eGFR) and osteoprotegerin (OPG) for cardiovascular (CVD) and all-cause mortality risk have been presented by our group and others. We now present data on the interactions between OPG with stage I to III chronic kidney disease (CKD) for all-cause and CVD mortality.

**Methods and Results:**

The setting was a 15-year study of 1,292 women over 70 years of age initially randomized to a 5-year controlled trial of 1.2 g of calcium daily. Serum OPG and creatinine levels with complete mortality records obtained from the Western Australian Data Linkage System were available. Interactions were detected between OPG levels and eGFR for both CVD and all-cause mortality (P < 0.05). Compared to participants with eGFR ≥60ml/min/1.73m^2^ and low OPG, participants with eGFR of <60ml/min/1.73m^2^ and elevated OPG had a 61% and 75% increased risk of all-cause and CVD mortality respectively (multivariate-adjusted HR, 1.61; 95% CI, 1.27-2.05; P < 0.001 and HR, 1.75; 95% CI, 1.22-2.55; P = 0.003). This relationship with mortality was independent of decline in renal function (P<0.05). Specific causes of death in individuals with elevated OPG and stage III CKD highlighted an excess of coronary heart disease, renal failure and chronic obstructive pulmonary disease deaths (P < 0.05).

**Conclusion:**

The association between elevated OPG levels with CVD and all-cause mortality was more evident in elderly women with poorer renal function. Assessment of OPG in the context of renal function may be important in studies investigating its relationship with all-cause and CVD mortality.

## Introduction

An inverse association between estimated glomerular filtration rate (eGFR) and cardiovascular disease (CVD) and all-cause mortality has been established in several populations [1, 2], such that individuals with and eGFR of <60 ml/min/1.73m^2^ [stage III chronic kidney disease (CKD)] have at least a 20% greater risk of CVD and all-cause mortality compared to those with an eGFR of ≥60 ml/min/1.73m^2^. Similarly rapid renal decline, defined as an annual reduction in eGFR of > 3 ml/min/1.73m^2^ is associated with a 2-fold increase risk of atherosclerotic vascular disease mortality further emphasizing the relationship between renal dysfunction and CVD risk [3, 4].

Osteoprotegerin (OPG) is a glycoprotein that has been shown to regulate several important organ systems by inhibiting the interaction between receptor activator of nuclear factor kappa-B ligand (RANKL) and its receptor RANK [5–7]. Other than its key role in the regulation of bone resorption, the OPG/RANKL/RANK system is involved in the pathogenesis of endothelial dysfunction, vascular inflammation [8] and vascular calcification [9–11]. We have demonstrated an independent association between elevated serum OPG levels and increased 8.5 year risk of CVD and all-cause mortality in elderly women [12], while others have demonstrated similar associations in patients with diabetes [10], severe renal dysfunction [13] and renal transplant patients [14, 15], and in those with pre-existing CVD [9, 12, 16].

While circulating OPG levels are increased in CKD patients [17], possibly related to reduced clearance, it remains unclear whether the association between OPG and the risk of vascular and all-cause mortality is modified by this declining renal function. We recently demonstrated elevated OPG levels were independently associated with a more rapid decline in renal function and the development of CKD in this cohort of elderly women with stages I-III CKD [18] suggesting a mechanism for the association of OPG with mortality.

We therefore sought to investigate both the pathophysiology and the predictive value of elevated OPG levels in elderly women with mild to moderate renal dysfunction.

## Methods

### Ethics statement

At baseline written informed consent was obtained from all participants for the study and follow up of electronic health records. The Human Ethics Committee of the University of Western Australia approved the study protocol and consent form (approval number 05/06/004/H50). The Human Research Ethics Committee of the Western Australian Department of Health (DOHWA HREC) also approved the data linkage study (approval number #2009/24).

### Study population

The participants were recruited in 1998 to a 5-year prospective, randomised, controlled trial of oral calcium supplements to prevent osteoporotic fractures, the Calcium Intake Fracture Outcome study (CAIFOS) [19]. The RCT was commenced and completed before the introduction of clinical trial registries as such no clinical trial registration number exists. Women were recruited from the Western Australian general population of women aged over 70 years by mail using the electoral roll, which is a requirement of citizenship. Of the 5,586 women approached, 1,500 women were recruited into the study. All participants were ambulant with an expected survival beyond 5 years and were not receiving any medication (including hormone replacement therapy) known to affect bone metabolism. Baseline disease burden and medications were comparable between these participants and the general population of similar age although these participants were more likely to be from higher socio-economic groups [19]. In the subsequent 5 years following inclusion in the study, participants had received 1.2 g of elemental calcium as calcium carbonate daily or matching placebo. At the conclusion of CAIFOS, participants were subsequently included in a 10 year observational follow-up.

### Baseline risk factors

Baseline medical history including the presence of diabetes, hypertension, smoking history (current smokers/former smokers or non-smokers) and medication use were recorded. Participant’s previous medical history and current medications were verified by their General Practitioner where possible. These data were coded using the International Classification of Primary Care—Plus (ICPC-Plus) method [20]. The coding methodology allows aggregation of different terms for similar pathologic entities as defined by the ICD-10 coding system. These data were then used to determine the presence of pre-existing diabetes (T89001-90009). Cardiovascular medications included beta-blockers, angiotensin-converting enzyme inhibitors, angiotensin II receptor blockers, 3-hydroxy-3-methylglutaryl-coenzyme A (HMG-Co A) reductase inhibitors and anti-platelet agents (Aspirin or Clopidogrel). Weight was obtained using digital scales with participants wearing only light clothing and without shoes, height was measured using a stadiometer and the body mass index (BMI) was calculated by weight (kg) / height^2^ (meters). Smoking status was coded as non-smoker or ex-smoker/current smoker if they had consumed more than 1 cigarette per day for more than 3 months at any stage during their lifetime. Blood pressure was measured on the right arm with a mercury column manometer using an adult cuff after the participants have been seated in an upright position and had rested for 5 minutes. An average of three blood pressure readings was recorded. Mean arterial pressure (MAP) was calculated using the following equation = [(2 x diastolic blood pressure) + systolic blood pressure] / 3.

### Biochemistry

Fasting blood samples for biochemistry were collected in 1998 and 2003 with sera stored at -80°C until analysis. Free OPG was measured in 2005 using the baseline sera from 1,333 (89%) participants using a validated enzyme immunoassay (R&D Systems, Minneapolis, MN, USA) as previously described [21, 22]. The reported intra- and inter-assay coefficients of variation of the immunoassays were 3.6% and 10.6% respectively [22]. In 2005, creatinine was measured using baseline fasting sera stored at -80°C using an isotope dilution mass spectrometry (IDMS) traceable Jaffe kinetic assay for creatinine on a Hitachi 917 analyzer (Roche Diagnostics GmbH, Mannheim Germany). Five-year creatinine was measured on the Architect ci16200 analyzer (Abbott, Illinois, U.S.A). The correlation coefficient (r^2^) between the machines was 0.998 with a Passing and Bablok slope of 0.966 and a Passing and Bablok intercept of 6.16 (n = 37) as described previously [18]. Estimated glomerular filtration rate (eGFR) was calculated using the Chronic Kidney Disease Epidemiology Collaboration (CKD-EPI) equations: 1) for standardized serum creatinine ≤ 0.7 mg/dL eGFR = 144 × (Scr / 0.7)^-0.329^ × (0.993)^Age^, 2) for standardized serum creatinine > 0.7 mg/dL eGFR = 144 × (Scr / 0.7)^-1.209^ × (0.993) ^Age^ [23].

### Prevalent disease

Prevalent disease status were derived from the International Classification of Diseases, Injuries and Causes of Death Clinical Modification (ICD-9-CM) [24] from 1980–1998 (baseline) for renal and cardiovascular disease. All health records were obtained from the Western Australian Data Linkage System (WADLS), which is a comprehensive, population-based linkage system connecting 40 years of clinical data from over 30 health related datasets for Western Australian residents using ICD codes [25]. Prevalent renal disease codes included glomerular diseases (codes 580–583); renal tubulo-interstitial diseases (593.3–593.5, 593.7 and 590–591); renal disease (codes 584–586); and hypertensive renal disease (code 403). Prevalent coronary heart disease (ICD-9-CM codes 410–414); heart failure (ICD-9-CM code 428) and cerebrovascular disease excluding haemorrhage (ICD-9-CM codes 433–438). A comorbidity score (1–7) was calculated from history of coronary heart disease, cerebrovascular disease, heart failure, diabetes, renal disease, treatment for dyslipidaemia, and hypertension based on blood pressure and/or treatment for hypertension as recommended by the 7^th^ Report of the Joint National Committee on Prevention, Detection, Evaluation, and Treatment of High Blood Pressure [26].

### Mortality outcomes

Mortality records were obtained from WADLS for each study participant between 1998 and 2013. International Classification of Diseases, Injuries and Causes of Death (ICD) primary and multiple cause of death were determined from the coded death certificate using information in Parts 1 and 2 of the death certificate or all diagnosis text fields from the death certificate where ICD 10 coded death data were not yet available. Deaths were defined using diagnosis codes from the ICD: Clinical Modification (ICD-9-CM) [24] and the International Statistical Classification of Diseases and Related Health Problems, 10th Revision, Australian Modification (ICD-10-AM) [27]. Primary cause of death codes included cardiovascular disease (ICD-9-CM codes 390–459 and ICD-10-AM codes I00-I99); cancer deaths (ICD-9-CM code 140–239 excluding 210–229 and ICD-10-AM code C00-D48 excluding D10-D36) and other deaths (all other codes).

### Statistical analysis

Baseline characteristics are presented as mean ± SD for continuous variables or median and interquartile range (IQR) for non-normally distributed variables. OPG was not normally distributed and was log transformed for analyses. OPG levels were categorised as above and below median cut-point of 2.2ng/mL. Effect modification between covariates and elevated OPG with vascular and all-cause mortality was examined by interaction tests with significant interactions detected using Cox regression. Participants were then categorised into 4 groups according to their OPG levels (above the median; 2.2 ng/mL—elevated, below the median—low) and eGFR measured by CKD-EPI eGFR (≥ 60 mL/min/1.73m2 and < 60ml/min/1.73m2). Models adjusting for 5-year change in eGFR excluded individuals with loss to follow-up due to withdrawal from the study and/or death or no measurement of 5-year creatinine (n = 325). Unadjusted and multivariable- adjusted Cox regression analyses were undertaken using IBM SPSS Statistics Version 21 (2012, Armonk, NY: IBM Corp). No violations of the Cox proportional hazards assumptions were detected. To exclude the possibility of reverse causality additional analyses were undertaken excluding participants who died within the first 24 months. Multivariable-adjustments included baseline age, body mass index, smoking history, treatment code (calcium or placebo), hormone replacement therapy and comorbidity score. P-values of less than 0.05 in two tailed testing were considered statistically significant.

## Results

### Interaction between OPG and eGFR

**Fig 1:**
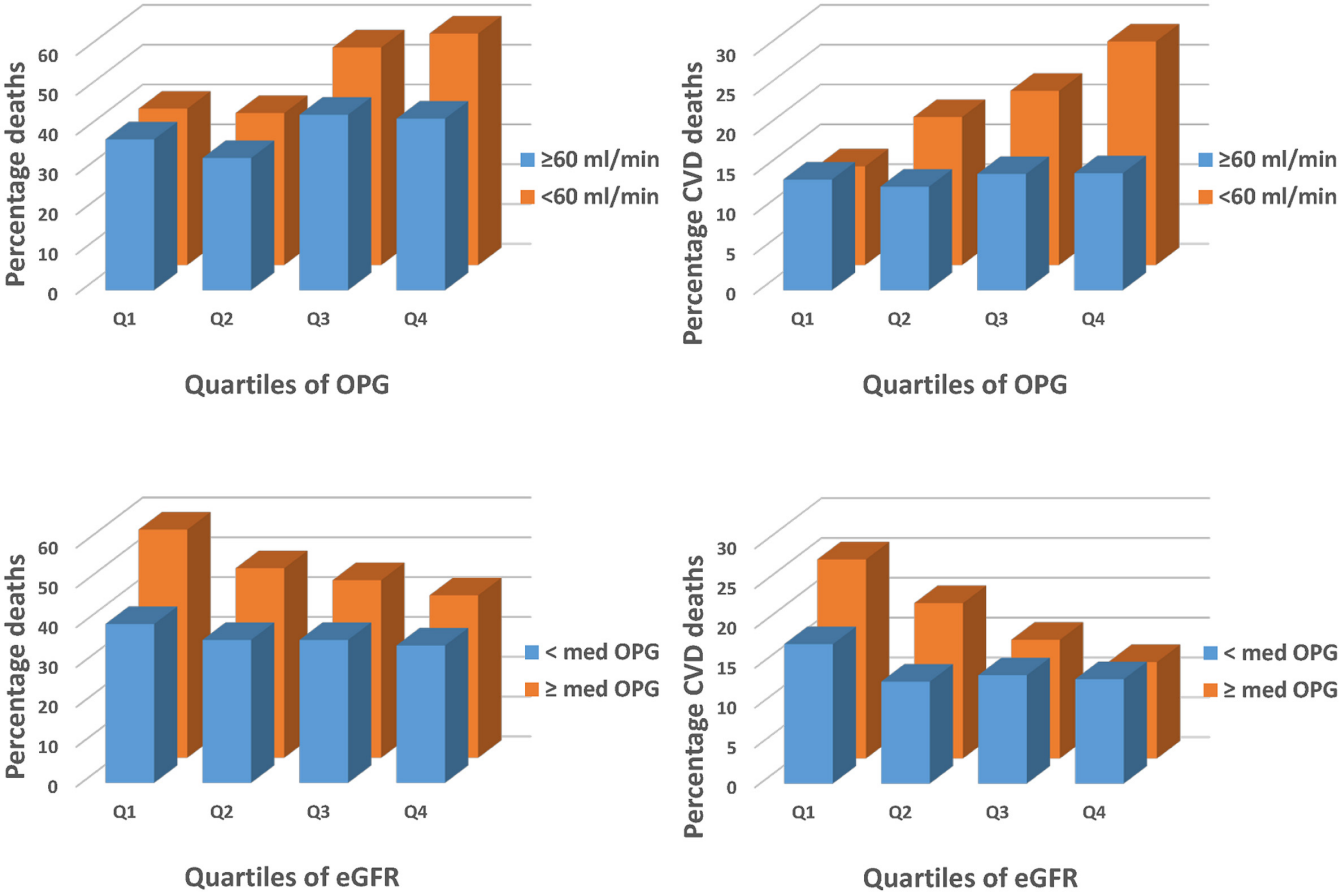
Relationship between quartiles of circulating OPG dichotomized by eGFR categories (≥ 60ml/min/1.73m^2^ and < 60ml/min/1.73m^2^) for all-cause mortality (top left, n = 547) and cardiovascular mortality (top right, n = 210) and relationship between quartiles of estimated glomerular filtration rate by the CKD-EPI equation (CKD-EPI eGFR) dichotomized by circulating OPG levels (< median and ≥ median) for all-cause mortality (bottom left) and cardiovascular mortality (bottom right).

As we sought to determine whether eGFR modified or accounted for the previously observed relationship between OPG and mortality, interaction tests between OPG and eGFR were undertaken. Using both variables as continuous (log transformed OPG and eGFR ml/min/1.73m^2^) there was an interaction observed between OPG and eGFR for all-cause mortality (P = 0.044) and CVD mortality (P = 0.028). Similarly when eGFR was dichotomized into above or below stage 3 CKD (eGFR < 60 ml/min/1.73m^2^) there was a significant interaction with log transformed OPG and stage of CKD for all-cause mortality (P = 0.043) and CVD mortality (P = 0.016) or when dichotomizing by median OPG levels with eGFR (ml/min/1.73m^2^) the interaction term remained significant for all-cause (P = 0.021) and CVD mortality (P = 0.047). Accordingly the participants were stratified into 4 groups according to median OPG levels (<2.2 ng/mL and ≥2.2 ng/mL) and eGFR dichotomised by the presence or absence of stage III CKD (< and ≥60mL/min/1.73m^2^). Graphical representation of these interactions are presented in Fig 1.

### Participant characteristics

Baseline characteristics of the four groups are presented in Table 1. Participants with an eGFR CKD-EPI < 60 ml/min/1.73m^2^ were older, had a higher BMI, more likely to be hypertensive, had a greater prevalence of renal disease and cardiovascular disease and were more likely to be prescribed statins or low dose aspirin at baseline (P < 0.05). Participants with elevated circulating OPG levels were older, more likely to be hypertensive, had a greater prevalence of diabetes and were more likely to be taking low dose aspirin at baseline.

**Table 1:** General characteristics of the study population.

	Low OPG eGFR ≥60	Elevated OPG eGFR ≥60	Low OPG eGFR <60	Elevated OPG eGFR <60
**Number**	**459**	**414**	**191**	**228**
**Osteoprotegerin, ng/mL**	1.8 [1.5–2.0]	2.7 [2.4–3.1]	1.8 [1.6–2.0]	2.8 [—2.0]
**CKD-EPI eGFR, mL/min/1.73m** ^**2**^	74.1 ± 8.6	73.2 ± 8.7	52.1 ± 6.4	50.9 ± 7.1
**Age, years** *	74.6 ± 2.5	75.3 ± 2.6	75.2 ± 2.7	76.1 ± 3.0
**Body mass index, kg/m** ^**2**^	27.2 ± 4.4	26.8 ± 4.6	27.5 ± 4.3	27.7 ± 5.5
**Calcium treatment, yes (%)**	242 (52.7)	224 (54.1)	81 (42.9)	123 (53.9)
**Smoking, yes (%)**	170 (37.2)	151 (36.7)	78 (40.8)	76 (33.8)
**Disease history**				
**Diabetes, yes (%)** *	20 (4.4)	34 (8.2)	11 (5.8)	22 (9.6)
**Renal disease, yes (%)** *	2 (0.4)	5 (1.2)	6 (3.1)	10 (4.4)
**Coronary heart disease, yes (%)** *	32 (7.0)	32 (7.7)	20 (10.5)	34 (14.9)
**Ischemic cerebrovascular disease, yes (%)** *	5 (1.1)	13 (3.1)	10 (5.2)	13 (5.7)
**Heart failure, yes (%)**	2 (0.5)	1 (0.2)	0 (0.0)	1 (0.4)
**History of hormone replacement therapy, yes (%)**	74 (16.2)	65 (15.8)	34 (17.8)	31 (13.7)
**Blood Pressure** ^†^				
**Systolic blood pressure, mmHg** *	136.2 ± 17.0	139.7 ± 18.5	138.0 ± 20.5	138.5 ± 17.1
**Diastolic blood pressure, mmHg**	72.4 ± 10.3	73.6 ± 10.6	73.9 ± 12.0	72.8 ± 11.6
**Mean arterial pressure, mmHg**	93.6 ± 10.9	95.7 ± 11.7	95.2 ± 13.5	94.7 ± 11.8
**Lipid profiles** ^‡^				
**Total cholesterol, mmol/L**	226 ± 43	226 ± 41	226 ± 40	231 ± 46
**Low density lipoprotein cholesterol, mmol/L**	141 ± 38	143 ± 38	141 ± 37	145 ± 43
**High density lipoprotein cholesterol, mmol/L** *	58 ± 15	56 ± 14	54 ± 13	54 ± 14
**Triglycerides, mmol/L** *	131 ± 64	134 ± 56	156 ± 77	155 ± 71
**Medication use**				
**Anti-hypertensives, yes (%)** *	164 (35.7)	166 (40.1)	93 (48.7)	129 (56.6)
**Statins, yes (%)** *	71 (15.5)	80 (19.3)	46 (24.1)	49 (21.5)
**Low dose aspirin, yes (%)** *	171 (15.5)	93 (22.5)	42 (22.0)	68 (29.5)
**Comorbidity score, (1–7)** *	1 [0–1.0]	1 [0–2.0]	1 [0–2.0]	1 [0–2.0]

Data expressed as mean ± SD or number and (%). Abbreviations: OPG, osteoprotegerin; CKD, chronic kidney disease; mmHg, millimeters mercury; CKD-EPI eGFR, Chronic Kidney Disease Epidemiology Collaboration estimated glomerular filtration rate.

* Significantly different between groups by ANOVA or χ^2^ test where appropriate (P<0.05)

^†^ measured in 1,252 participants and

^‡^ measured in 974 participants.

### All-cause mortality

In participants with an eGFR <60mL/min/1.73m^2^ (n = 419) per SD increase in OPG there was a 28% increase in the risk of 15 year death in unadjusted (HR 1.28, 95%CI 1.13–1.46, P<0.001) that remained significant after multivariable-adjustment (HR 1.25, 95%CI 1.09–1.43, P = 0.001). In participants with an eGFR ≥60mL/min/1.73m^2^ per SD increase in OPG there was a marginally non-significant 11% increase in the risk of 15 year death in unadjusted (HR 1.11, 95%CI 1.00–1.22, P = 0.055) that remained non-significant after multivariable-adjustment (HR 1.05, 95%CI 0.94–1.17, P = 0.398).

Participants with below-median OPG levels and eGFR ≥60mL/min/1.73m^2^ had the lowest incidence of all-cause mortality (35.3%) and therefore were designated the referent group.

**Fig 2:**
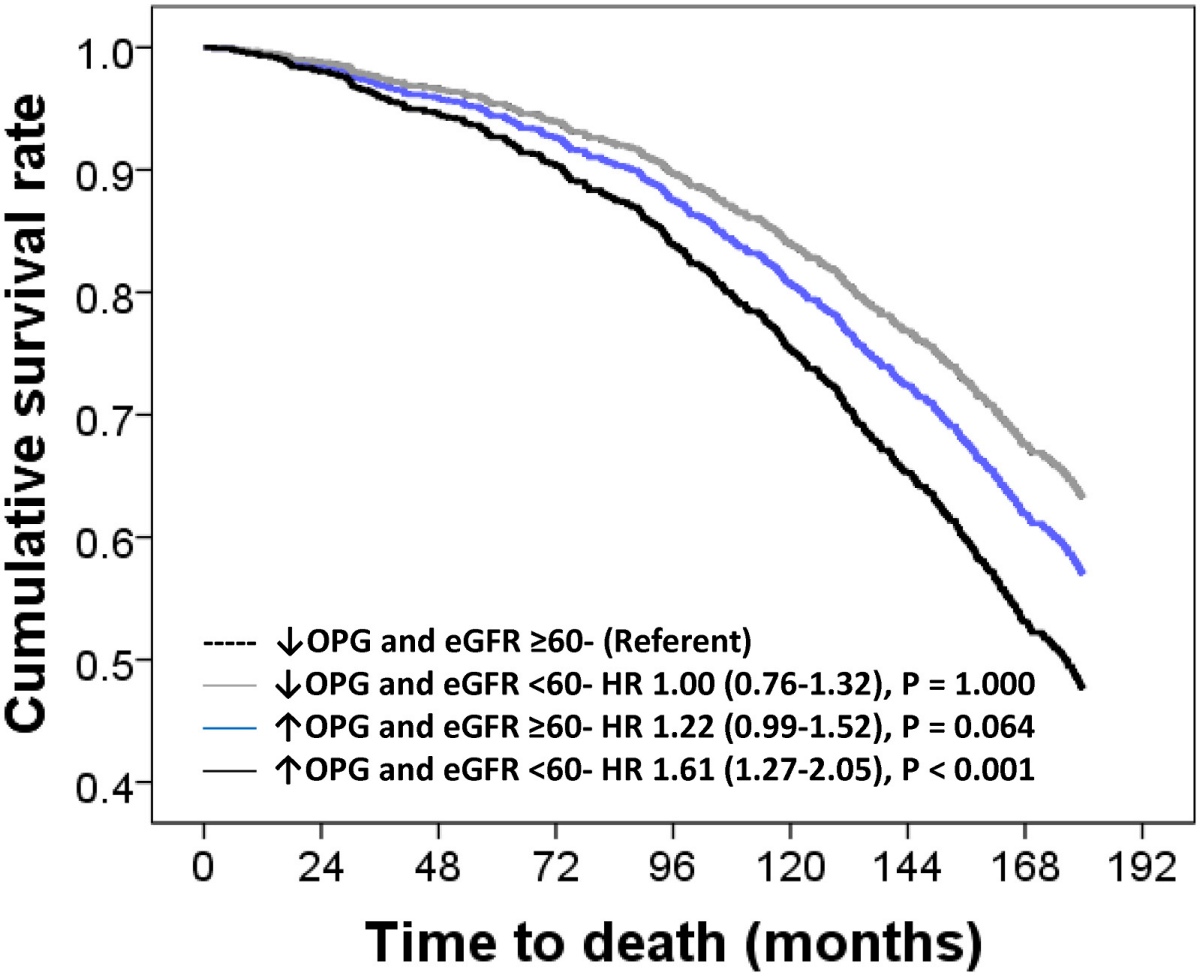
Multivariable-adjusted hazard ratio (HR) and 95% confidence interval for 15-year all-cause mortality (n = 547) in participants dichotomized by OPG levels and eGFR. Multivariable-adjustments were baseline age, body mass index, smoking history, history of hormone replacement therapy, treatment code (calcium or placebo) and comorbidity score.

Participants with above-median OPG levels and eGFR ≥60mL/min/1.73m^2^ had significantly higher incidence of 15-year all-cause mortality (44.0%) in unadjusted models (HR 1.35, 95%CI 1.09–1.66, P = 0.006) and that became non-significant after multivariable adjustment (Fig 2). Compared to participants with below-median OPG levels and eGFR ≥60mL/min/1.73m^2^, participants with elevated OPG above the median and an eGFR <60mL/min/1.73m^2^ had the highest incidence of all-cause mortality (56.6%) before adjustment (HR 1.97, 95%CI 1.56–2.48, P < 0.001) that remained significant after adjustment (Fig 2). Participants with below the median OPG levels and eGFR ≥60mL/min/1.73m^2^ were not at a higher risk in before adjustment (HR 1.13, 95%CI 0.85–1.48, P = 0.401) or after multivariate-adjusted analyses (Fig 2).

### Primary cause of death data (PCoD)

In participants with an eGFR <60mL/min/1.73m^2^ (n = 419) per SD increase in OPG there was a 43% increase in the risk of 15 year cardiovascular death in unadjusted (HR 1.43, 95%CI 1.19–1.71, P<0.001) that remained significant after multivariable-adjustment (HR 1.39, 95%CI 1.14–1.70, P = 0.001). In participants with an eGFR ≥60mL/min/1.73m^2^ per SD increase in OPG there was no increase in the risk of 15 year cardiovascular death in unadjusted (HR 1.06, 95%CI 0.89–1.26, P = 0.523) that remained non-significant after multivariable-adjustment (HR 1.00, 95%CI 0.83–1.12, P = 0.974).

**Fig 3:**
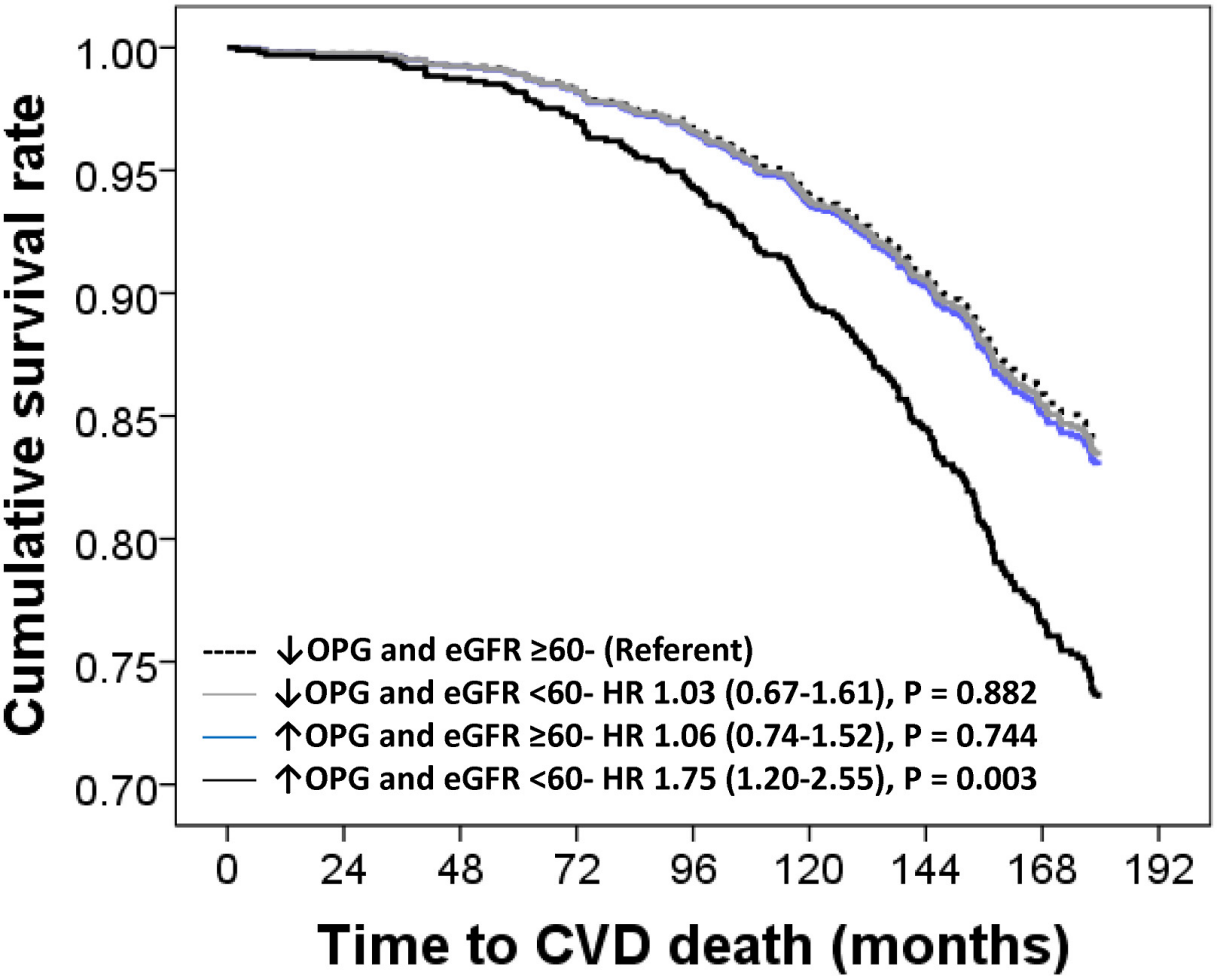
Multivariable-adjusted hazard ratio (HR) and 95% confidence interval for 15-year cardiovascular mortality (n = 210) in participants dichotomized by baseline OPG levels and eGFR. Multivariable-adjustments were baseline age, body mass index, smoking history, history of hormone replacement therapy, treatment code (calcium or placebo) and comorbidity score.

Compared to participants with below-median OPG levels and eGFR ≥60mL/min/1.73m^2^, only participants with above-median OPG levels and eGFR <60mL/min/1.73m^2^ had significantly higher risk of 15-year CVD mortality before (HR 2.33, 95%CI 1.62–3.33, P < 0.001) and after adjustment for age, BMI, treatment, smoking history and prevalent renal disease and diabetes (Fig 2). Participants with below-median OPG levels and eGFR ≥60mL/min/1.73m^2^ and above-median OPG with eGFR ≥60mL/min/1.73m^2^ were not at a higher risk of CVD mortality in unadjusted (HR 1.19, 95%CI 0.77–1.85, P = 0.431 and HR 1.19, 95%CI 0.83–1.69, P = 0.343 respectively) or multivariate analyses (Fig 3).

### Cause of Death (MCoD) data (Table 2)

Unlike the primary cause of CVD death data participants with above-median OPG levels and eGFR ≥60mL/min/1.73m^2^ and an eGFR of <60mL/min/1.73m^2^ had increased risk of CVD-related deaths compared to participants with below-median OPG levels and eGFR ≥60mL/min/1.73m^2^ before (HR 1.46, 95%CI 1.11–1.91, P = 0.006 and HR 2.45, 95%CI 1.84–3.25, P < 0.001 respectively) and after multivariable adjustment (Table 3). Adverse outcomes in participants with elevated OPG levels were only observed for CHD and COPD-related mortality in participants with an eGFR of <60 ml/min/1.73m^2^ in multivariable-adjusted models (Table 3).

**Table 2:** 15-year cause of death.

	Low OPG eGFR ≥60	Elevated OPG eGFR ≥60	Low OPG eGFR <60	Elevated OPG eGFR <60	P value
**Number of participants**	**459**	**414**	**191**	**228**	
**Number of deaths** *	**162 (35.3)**	**182 (44.0)**	**74 (38.7)**	**129 (56.6)**	**<0.001**
**Primary cause of death (PCoD)**					
**CVD, No. (%)**	**62 (13.5)**	**61 (14.8)**	**30 (15.7)**	**57 (27.2)**	**0.001**
**Cancer, No. (%)**	51 (11.1)	54 (13.1)	16 (8.4)	26 (11.5)	0.405
**“Other”, No. (%)**	**49 (10.7)**	**67 (16.2)**	**28 (14.7)**	**46 (20.2)**	**0.007**
**Multiple cause of death (MCoD)**					
**CVD, No. (%)**	**96 (20.9)**	**116 (28.1)**	**49 (25.7)**	**93 (41.2)**	**<0.001**
**CHD, No. (%)**	**47 (10.2)**	**50 (12.1)**	**25 (13.1)**	**54 (23.9)**	**<0.001**
**Ischemic cerebrovascular disease, No. (%)**	41 (8.9)	26 (6.3)	19 (9.9)	26 (11.5)	0.130
**Peripheral arterial disease, No. (%)**	6 (1.3)	5 (1.2)	2 (1.0)	6 (2.7)	0.441
**Heart failure, No. (%)**	22 (4.8)	33 (8.0)	9 (4.7)	19 (8.4)	0.106
**Hypertension, No. (%)**	34 (7.4)	43 (10.4)	17 (8.8)	27 (11.9)	0.220
**Renal failure, No. (%)**	**11 (2.4)**	**22 (5.3)**	**10 (5.2)**	**24 (10.6)**	**<0.001**
**Cancer, No. (%)**	65 (14.2)	58 (14.0)	18 (9.4)	38 (16.8)	0.184
**Dementia, No. (%)**	31 (6.8)	33 (8.0)	14 (7.3)	28 (12.4)	0.081
**Diabetes, No. (%)**	15 (3.3)	21 (5.1)	4 (2.1)	12 (5.3)	0.197
**Respiratory, No. (%)**	**52 (11.3)**	**60 (14.5)**	**17 (8.9)**	**41 (18.1)**	**0.019**
**COPD**	**25 (5.4)**	**33 (8.0)**	**9 (4.7)**	**25 (11.1)**	**0.025**
**“Other respiratory” excluding COPD**	27 (5.9)	27 (6.5)	8 (4.2)	16 (7.1)	0.619
**Infectious and parasitic, No. (%)**	10 (2.2)	14 (3.4)	4 (2.1)	5 (2.2)	0.641

Data expressed as mean ± SD or number and (%). P value represents overall P value for the trend by χ^2^ test. Abbreviations: OPG, osteoprotegerin; CKD, chronic kidney disease; CVD cardiovascular disease; CHD coronary heart disease, COPD, chronic obstructive pulmonary disease.

*Disease specific multiple cause of deaths numbers do not add up to total mortality as multiple causes of death are possible.

**Table 3:** Multivariable-adjusted hazard ratio for deaths using multiple-cause of death data according to circulating OPG levels and kidney function.

	No. (%)	HR (95%CI)*	P value
**Total CVD**			
**Low OPG + eGFR ≥60 mL/min/1.73m** ^**2**^	96/459 (20.9)	1.00 (reference)	
**Elevated OPG + eGFR ≥60 mL/min/1.73m** ^**2**^	116/414 (28.0)	1.27 (0.97–1.67)	0.086
**Low OPG + eGFR <60 mL/min/1.73m** ^**2**^	49/191 (25.7)	1.06 (0.75–1.50)	0.743
**Elevated OPG + eGFR <60 mL/min/1.73m** ^**2**^	**93/228 (40.8)**	**1.84 (1.37–2.48)**	**<0.001**
**CHD**			
**Low OPG + eGFR ≥60 mL/min/1.73m** ^**2**^	47/459 (10.2)	1.00 (reference)	
**Elevated OPG + eGFR ≥60 mL/min/1.73m** ^**2**^	50/414 (12.1)	1.09 (0.73–1.63)	0.690
**Low OPG + eGFR <60 mL/min/1.73m** ^**2**^	25/191 (13.1)	1.04 (0.64–1.71)	0.864
**Elevated OPG + eGFR <60 mL/min/1.73m** ^**2**^	**54/228 (23.7)**	**2.07 (1.38–3.11)**	**<0.001**
**Renal failure**			
**Low OPG + eGFR ≥60 mL/min/1.73m** ^**2**^	11/459 (2.4)	1.00 (reference)	
**Elevated OPG + eGFR ≥60 mL/min/1.73m** ^**2**^	**22/414 (5.3)**	**2.12 (1.02–4.40)**	**0.043**
**Low OPG + eGFR <60 mL/min/1.73m** ^**2**^	10/191 (5.2)	1.81 (0.76–4.31)	0.179
**Elevated OPG + eGFR <60 mL/min/1.73m** ^**2**^	**24/228 (10.5)**	**4.12 (1.98–8.55)**	**<0.001**
**COPD**			
**Low OPG + eGFR ≥60 mL/min/1.73m** ^**2**^	25/459 (5.4)	1.00 (reference)	
**Elevated OPG + eGFR ≥60 mL/min/1.73m** ^**2**^	33/414 (8.0)	1.43 (0.84–2.42)	0.185
**Low OPG + eGFR <60 mL/min/1.73m** ^**2**^	9/191 (4.7)	0.79 (0.37–1.70)	0.540
**Elevated OPG + eGFR <60 mL/min/1.73m** ^**2**^	**25/228 (11.0)**	**2.21 (1.25–3.93)**	**0.007**

Abbreviations: OPG, osteoprotegerin; HR, hazard ratio; Total CVD, total cardiovascular disease; CHD, coronary heart disease; COPD, chronic obstructive pulmonary disease.

*Multivariable-adjustments were baseline age, body mass index, smoking history, history of hormone replacement therapy, treatment code (calcium or placebo) and comorbidity score.

### Potential mechanism(s)

**Fig 4:**
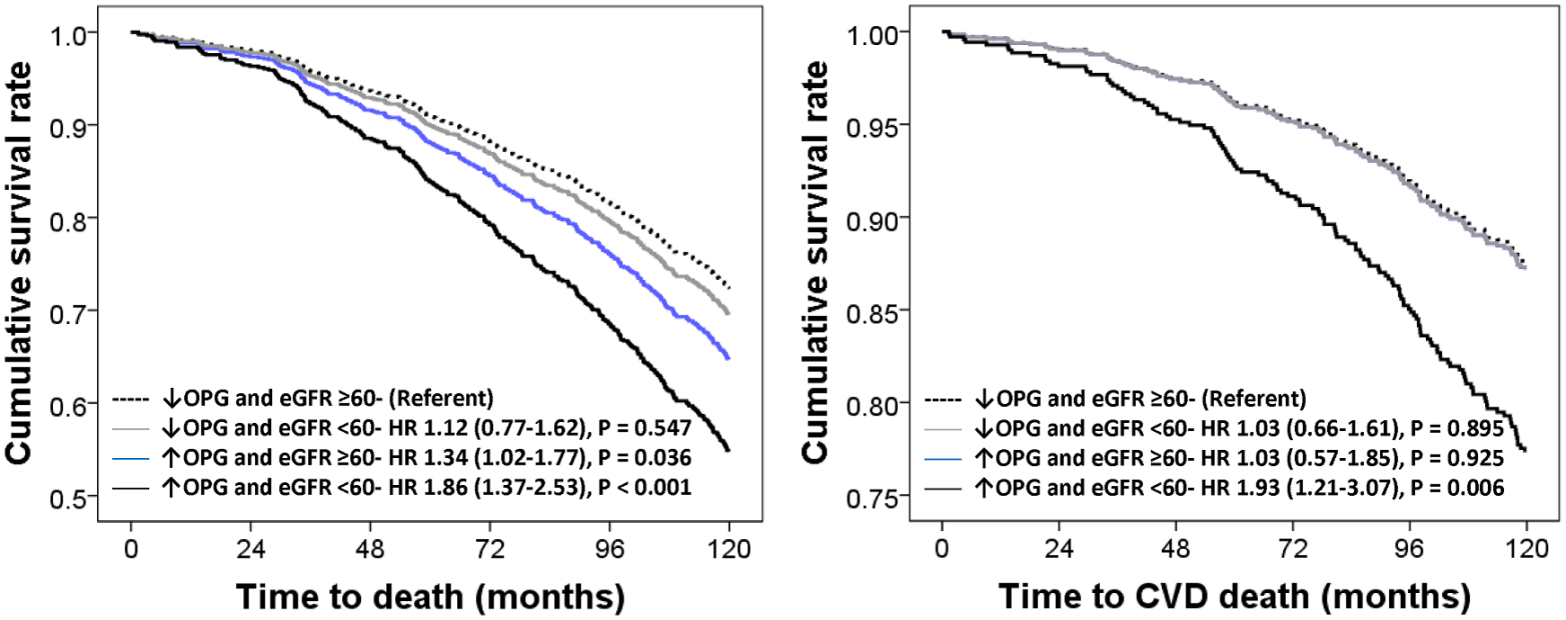
Multivariable model plus five-year change in eGFR-adjusted hazard ratio (HR) and 95% confidence interval for 10-year (2003–2013) all-cause (n = 339) and cardiovascular mortality (n = 135) in participants dichotomized by baseline OPG levels and eGFR. Multivariable-adjustments included 5-year change in CKD-EPI eGFR (n = 970), age, body mass index, smoking history, history of hormone replacement therapy, treatment code (calcium or placebo) and comorbidity score.

To assess whether the relationship between circulating OPG levels with mortality outcomes was attributed to long-term renal decline, a Cox regression model including the 5-year change in eGFR was created (Fig 4). For all-cause and CVD mortality, the association between elevated OPG levels and CKD remained unchanged. Five-year change in eGFR was independently associated with both all-cause (n = 339) and CVD mortality (n = 134; P = 0.007 and P = 0.019 respectively).

### Sensitivity analyses

When excluding participants with CVD at baseline (n = 302) only participants with elevated OPG and an eGFR <60 ml/min/1.73m^2^ were at an increased risk of CVD death before and after multivariate-adjustment (HR 2.09–1.33–3.29, P = 0.001 and HR 1.59, 95%CI 1.00–2.54, P = 0.050 respectively). Participants with elevated OPG and an eGFR ≥60 ml/min/1.73m^2^ had an increased risk of all-cause mortality before multivariable-adjustment (HR 1.30–1.03–1.66, P = 0.037) but not after multivariable-adjustment (HR 1.20–0.94–1.54, P = 0.153) while those with elevated OPG and an eGFR <60 ml/min/1.73m^2^ were at an increased risk before and after multivariate-adjustment (HR 1.99–1.50–2.62, P < 0.001 and HR 1.65, 95%CI 1.23–2.19, P = 0.001 respectively). To assess potential reverse causality bias we excluded all deaths that occurred within the first 24 months of study with little change to the overall result (elevated OPG and ≥60 ml/min/1.73m^2^ HR 1.22, 95%CI 0.98–1.52, P = 0.069 and elevated OPG and <60 ml/min/1.73m^2^ HR 1.63, 95%CI 1.28–2.08, P < 0.001 respectively) and CVD mortality (elevated OPG and <60 ml/min/1.73m^2^ HR 1.75, 95%CI 1.20–2.55, P = 0.004).

## Discussion

In a cohort of community-based ambulant elderly women, the association between OPG levels and all-cause and cardiovascular mortality appeared to be modified by reduced kidney function. Women with above-median OPG levels and an eGFR <60mL/min/1.73m^2^ had a 71–88% increased risk of all-cause and cardiovascular mortality and a 4-fold increase in renal failure mortality compared to participants with below-median OPG levels and without moderate CKD. In addition to demonstrating an association between OPG and mortality in individuals with stage III CKD, this study raises important considerations of how OPG may be involved in the pathophysiological of CVD mortality. These findings extend upon the previous study by our group [12] that found elevated OPG was associated with 8.5 year all-cause and CVD mortality but did not include measures of renal function and did not have previous disease history from hospital records available. These long-term differences in the risk between participants with elevated circulating OPG was independent of the change in eGFR and may be important in identifying individuals with a poorer long-term prognosis. The utility of OPG as a novel biomarker of participants with poorer clinical prognosis is enhanced by its low intra-individual variation, its relatively high levels in circulation and stability when exposed to freeze thaw and room temperature [28].

The increased mortality risk identified in individuals with elevated OPG levels appeared to diverge between 3 to 5 years after circulating OPG assessment and continued to diverge until the end of follow-up, suggesting a single measurement of circulating OPG levels may potentially help identify individuals with progressive disease that could result in death over a 15-year period [18]. This increased risk people with impaired renal function is in good accord with other studies of patients with stage 4 & 5 CKD and haemodialysis patients that found elevated OPG is strongly associated with all-cause and CVD mortality[13, 29]. Taken together these findings supports the concept that OPG may be an important biomarker in individuals with stage 3–5 CKD with a poorer long-term prognosis.

Regarding potential mechanisms, OPG is derived locally from both bone and vascular smooth muscle cells and is present in high concentrations throughout all layers of normal and atherosclerotic blood vessel walls [30]. Circulating osteoprotegerin levels have been suggested to increase with stage of CKD [31] and are consistently found to be related to vascular calcification in humans [11, 17, 31, 32]. Animal models support the concept that OPG is an inhibitor of vascular calcification but not atherosclerosis as mice treated with recombinant OPG had reduced calcified lesion area without affecting atherosclerotic lesion area [33]. Therefore high circulating levels of OPG may reflect a compensatory mechanism whereby injuries to the blood vessel wall result in the release of OPG from within the blood vessel wall into the circulation [34] or the injuries to blood vessel wall enhance OPG production during active calcification [29]. Our results support the concept that OPG levels are higher in participants with stage 3 CKD and may identify individuals with poorer outcomes particularly as renal function declines.

Although it has been suggested that the elevated OPG levels detected in CKD and ESRD patients are attributable to reduced renal clearance, it is also possible that an excess of production of OPG could also be contributory given its role in regulating calcification. However the rapid fall observed in OPG levels following successful renal transplantation in ESRD patients supports the former hypothesis [35]. Despite this, the association between OPG and mortality independent of baseline and 5-year change in eGFR as well as traditional CVD risk factors suggests that the association between elevated OPG and mortality is independent of declining renal function. Nevertheless in patients with less severe renal dysfunction it may also be that circulating OPG levels are an additional marker of vascular disease leading to further renal deterioration in addition to serum creatinine.

Using Multiple Cause of Death (MCoD) data, we were able to identify coronary artery disease as the major identifiable cause of increased cardiovascular disease mortality in participants with an eGFR <60/ml/min/1.73m^2^. The clinical manifestations of coronary artery disease include: myocardial infarction, angina and acute coronary syndrome and chronic coronary heart disease. Given the relationship between OPG and long-term renal decline observed in this cohort previously [18], the association between OPG and renal-failure mortality is likely related to its association with long-term renal decline seen in this cohort [18]. Interestingly, chronic obstructive pulmonary disease deaths were identified as a potential concurrent disease process supporting the findings of Eagan et al. [36] that OPG levels were higher in increasing severity of COPD stages. However it remains uncertain as to whether these elevated OPG is causal or merely be an epiphenomenon. Further study in this area of research is warranted.

There are several limitation of this study that must be considered, firstly these findings are only in elderly women and as such may not be generalizable to men and other younger age groups. A further limitation of the current study is that we did not test the interaction between OPG and other measures of chronic kidney disease such as albuminuria and did not adjust for other markers of inflammation, atherosclerosis or vascular calcification. Also as this was a prospective cohort study we lack data on the temporal sequence of the changes in OPG levels and the development of clinical events and thus cannot identify causality. However despite the lack of a temporal sequence of the change in circulating OPG we have demonstrated that OPG measured at a single time point when applied in the context moderate CKD identifies patients with a poorer clinical prognosis for up to 15 years in this cohort suggesting measuring OPG in individuals with CKD may improve identification of individuals with a poorer prognosis.

The strengths of this study include the complete and accurate data collection over a 15-year period independent of self-report in a large cohort of subjects and the ability to accurately examine the association between baseline OPG levels and renal function with long-term clinical outcomes removing the possibility of recall or retention bias. This comprehensive outcome data in conjunction with the measurement of both circulating OPG and creatinine has allowed the identification of the interaction between renal dysfunction and elevated OPG with CVD and all-cause mortality. In addition the use of multiple cause of death data which highlights relationships between concurrent disease processes and allows patterns of pathological damage to be elucidated is a strength of the study and has identified both renal failure and chronic obstructive pulmonary disease as potential mechanisms underlying the association between OPG and all-cause mortality.

In conclusion our findings supports the concept that the increased CVD and all-cause mortality risk associated with elevated OPG levels is primarily seen in individuals with moderate CKD. Measuring circulating OPG levels in individuals with at least moderate CKD may allow early identification of those at higher risk of CVD and all-cause mortality.
